# Pushing the boundaries - perioperative risk assessment and short-term surgical outcomes in octogenarian patients with head and neck cancer: comparison with non-oncological elderlies

**DOI:** 10.3389/fonc.2026.1748506

**Published:** 2026-03-12

**Authors:** Małgorzata Wierzbicka, Dorota Świątek, Maria Makuszewska, Kazimierz Niemczyk, Karolina Dżaman, Katarzyna Czerwaty, Bogusław Mikaszewski, Dominik Stodulski, Maciej Kawczyński, Magdalena Rękawek, Małgorzata Wierzchowska, Paweł Burduk, Kamila Sroka, Wioletta Pietruszewska, Katarzyna Ciuba, Jarosław Markowski

**Affiliations:** 1 Department of Otolaryngology, Regional Specialist Hospital Wrocław, Research & Development Centre, Wrocław, Poland; 2 Faculty of Medicine, Wrocław University of Science and Technology, Wrocław, Poland; 3 Institute of Human Genetics, Polish Academy of Sciences, Poznań, Poland; 4 Department of Otorhinolaryngology Head and Neck Surgery, Medical University of Warsaw, Warsaw, Poland; 5 Department of Otolaryngology, Centre of Postgraduate Medical Education, Warsaw, Poland; 6 Department of Otolaryngology, Faculty of Medicine, Medical University of Gdańsk, Gdańsk, Poland; 7 Department of Otolaryngology, Pomeranian Medical University, Szczecin, Poland; 8 Department of Otolaryngology, Phoniatrics and Audiology, Nicolaus Copernicus University in Torun, Collegium Medicum in Bydgoszcz, Bydgoszcz, Poland; 9 Department of Otolaryngology, Laryngological Oncology and Maxillofacial Surgery, University Hospital No2 in Bydgoszcz, Bydgoszcz, Poland; 10 Department of Otolaryngology, Phoniatrics and Audiology, Nicolaus Copernicus University, Bydgoszcz, Poland; 11 Department of Otolaryngology Head Neck Oncology, Medical University of Łódź, Łódź, Poland; 12 Department of Laryngology, Faculty of Medical Sciences in Katowice, Medical University of Silesia in Katowice, Katowice, Poland

**Keywords:** Caprini score, Charlson comorbidity index (CCI), elderly, geriatric oncology, head and neck cancer, multimorbidity, octogenarians, perioperative complications

## Abstract

**Introduction:**

There is an expanding body of research focuses on the clinical management of patients aged eighty and above. It remains uncertain whether therapeutic approaches for head and neck cancer in this demographic require a higher acceptance of perioperative risk compared to age-matched non-oncological individuals. This study aimed to characterize perioperative risk and early postoperative outcomes in octogenarian patients with and without head and neck cancer, and to explore how different geriatric and surgical risk scales perform in predicting adverse events in these two subgroups.

**Methods:**

This retrospective analysis encompassed data from eight university-affiliated otorhinolaryngology departments in Poland, covering the period from September 2023 to August 2024.

**Results:**

The oncological cohort exhibited a markedly higher prevalence of risk factors: male sex, smoking history, and previous malignancies compared with non-oncological cohort. Patients with head and neck cancer also demonstrated substantially elevated results on the Caprini, Charlson Comorbidity Index, American Society of Anesthesiologists Physical Status Classification, and Venous Thromboembolism scales, indicating increased multimorbidity and surgical risk. Although hospitalization durations were numerically longer for oncological cases, this difference was not statistically significant, and both groups showed similar functional independence and frailty scores. Logistic regression analyses highlighted that complication risk in non-oncological group was best predicted by Caprini scores, whereas the comorbidity index was the most informative predictor in oncological group. Cross-applied models failed to reach significance, confirming distinct risk patterns. Overall, sixteen percent of patients experienced at least one major complication, with oncological status independently associated with increased risk.

**Discussion:**

These findings demonstrate the multifactorial landscape of perioperative risk among the oldest surgical patients and provide quantitative evidence to support population-specific preoperative strategies in geriatric oncology.

## Introduction

Head and neck cancer (HNC) incidence and mortality have increased significantly among the elderly over the last two decades. This trend reflects the challenges of an aging population and underscores the need for tailored treatment strategies, particularly for the 80+ age group (1–3). There is a growing demand for specialized care to optimize treatment outcomes and ensure quality of life for elderly HNC patients (4–7).

Surgical treatment of HNC in octogenarians presents unique clinical challenges, primarily due to concerns over comorbidities, frailty, and increased risk of postoperative complications. Despite these concerns, recent studies demonstrate that chronological age alone should not be the sole determinant in treatment decisions; careful preoperative assessment and optimization can enable safe surgery with acceptable outcomes in well-selected octogenarian patients (8–10).

In large cohorts of patients 80+, overall postoperative complication rates are higher compared to younger elderly, with delirium, cardiovascular, and respiratory issues among the most frequent adverse events. Nevertheless, survival rates after surgical treatment, while lower than in younger groups, are comparable to age-specific life expectancy, and most patients recover to their previous level of independence (10–15).

There is a growing body of literature that addresses clinical guidelines for the management of patients aged 80 years and older. However, a critical and as yet unresolved question concerns whether the therapeutic approach to head and neck cancer in this population necessitates greater acceptance of perioperative and oncological risk compared to decision-making in age-matched individuals without malignancy. In this study, our primary objective was to characterize perioperative risk and early postoperative outcomes in octogenarian patients with and without head and neck cancer, and to examine how different geriatric and surgical risk scales perform in predicting adverse events in these two subgroups.

## Materials and methods

### Sample characteristics

The study was approved by the Bioethics Committee prior to data collection. This retrospective observational study included data collected over a 12-month period, from September 2023 to August 2024, across eight university-affiliated otorhinolaryngology departments in Poland (Wrocław (two hospitals), Gdańsk, Bydgoszcz, Szczecin, Warsaw, Katowice, and Łódź). No formal exclusion criteria were applied. All patients aged 80 years or older who were hospitalized in the participating otorhinolaryngology departments during the study period were eligible for inclusion. The sample consisted of 447 individuals aged 80–97 years. The subset of prolonged hospitalization (PH) included 49 out of 441 cases (with 6 cases of missing data), which equates to approximately 11% of the study population.

### Definition of variables and methodological assumptions

The primary outcome measures were selected to most accurately reflect the presence of risk factors among elderly patients: prolonged hospitalization (PH), the incidence of serious complications within 30 days, and functional decline within 90 days. PH was defined as a hospital stay exceeding two interquartile ranges (IQR) above the median inpatient duration. Given a median stay of three days and an IQR of three days, admissions lasting ten days or longer were classified as prolonged, in accordance with the economic criterion. Oncological family history was defined as the presence of any malignant tumor (including skin cancers such as squamous cell carcinoma) in first- or second-degree relatives.

Polypharmacy was defined as the concurrent, regular use of three or more prescription medications, a threshold widely adopted in geriatric and oncological research because of its established association with adverse drug events, falls, functional decline, and mortality in older adults (16).

Serious complications (30-days serious complications) were defined as postoperative adverse events occurring within 30 days of surgery that required at least pharmacological, interventional, or intensive care management, corresponding to Clavien–Dindo grade II or higher (e.g. events necessitating blood transfusion, total parenteral nutrition, reoperation, endoscopic or radiologic intervention, intensive care admission, or resulting in death) (17). In the present analysis, we did not stratify outcomes according to the receipt or timing of adjuvant radiotherapy, and 90-day functional decline was recorded irrespective of whether patients had initiated or completed radiotherapy during this interval.

Functional decline (90-days functional decline) was defined as a new or sustained deterioration in functional status within 90 days of index hospitalization, operationalised as a transition to a worse category in at least one of three domains-mobility, mode of feeding, or independence in basic and instrumental activities of daily living-resulting in increased need for assistance with everyday activities (18, 19).

Functional status, recognized as a fundamental determinant of prognosis in this population, was systematically assessed at the time of admission across three domains. Physical activity was categorized as independent, limited, or immobile, according to the patient’s capacity for unassisted ambulation. Feeding dependency was evaluated by direct observation, patient or caregiver interview, and review of medical records, using a four-point scale: independent oral intake (normal diet without assistance), oral intake requiring assistance or specialized preparation (for example, nutritarians), enteral feeding via gastrostomy, and enteral feeding via nasogastric tube. Independence in activities of daily living (ADL) was classified as independent (managing both basic and complex daily tasks), partially dependent (requiring assistance several times weekly for complex tasks), or fully dependent (requiring daily support for all basic activities). These data were collected and coded according to unified criteria across all eight participating centers to ensure methodological consistency and inter-site comparability.

In addition to the three variables representing functional status, the study incorporated gender, polypharmacy, alcohol consumption, smoking, oncological status (including malignancies located outside the head and neck region), and oncological family history.

The study incorporated six validated risk assessment tools, each with distinct clinical applications: ASA, CCI, Caprini, VTE, VES-13 and mFI-5. The ASA Physical Status Classification (I–VI) was recorded preoperatively by the attending anesthesiologist as a measure of global perioperative risk. The Charlson Comorbidity Index (CCI) is a weighted scale for assessing multimorbidity burden and mortality risk. In this study, patient comorbidities were identified through systematic review of the electronic medical record and clinical documentation at hospital admission. Caprini Venous Thromboembolism Risk Assessment Scale quantifies VTE risk based on patient demographic characteristics, comorbidities, and procedure type. According to the Caprini score, 0–1 points indicate low VTE risk, 2 points moderate risk, 3–4 points high risk, and ≥5 points very high risk. Vulnerable Elders Survey-13 (VES-13) is 13-item self-reported questionnaire assesses age, self-perceived health, functional limitations, and cognitive/physical performance. Scores ≥3 indicate high risk for functional decline. Frailty was additionally assessed with the mFI-5, which captures five domains (history of ischemic heart disease, hypertension, diabetes, chronic obstructive pulmonary disease, and impaired functional status). All measures were collected contemporaneously at hospital admission to ensure consistency of timing and to minimize recall bias, using a combination of structured patient interview and systematic review of the medical history and electronic medical records.

Group comparisons were conducted according to oncological status. For categorical or ordinal variables with three or fewer categories, the χ² test was applied. For continuous or ordinal variables with a larger number of categories, both Welch’s t-test for unequal variances (to assess mean differences) and the Mann-Whitney-Wilcoxon U test (to assess stochastic dominance) were used. Effect sizes were expressed as Cramer’s V for the χ² test, Cohen’s |d| for the t-test, and rank-biserial correlation for the U test. Given the number of comparisons performed on a single dataset, a conservative significance threshold of 0.01, or preferably 0.001, was applied. The analysis focuses on associations yielding p-values near or below this range.

To construct logistic regression models assessing which risk scales most effectively predict the occurrence of broadly defined complications, an artificial variable “Problems” was created, assigned a value of 1 when at least one of the following was present: prolonged hospitalization, 30-day serious complications, or 90-day functional decline, and a value of 0 when none of these issues were reported in the data. This variable took the value 1 for 16.3% of the cohort (n = 73), including 21.9% (46 out of 210) of oncological patients and 11.5% (26 out of 227) of non-oncological patients. The relationship between the “Problems” variable and oncological status was statistically significant (p = 0.003).

Separate logistic regression models were constructed to explain the risk of complications for oncological and non-oncological groups. Initially, for each subgroup, models included the relevant dependent variable and six explanatory variables representing the six risk scales under consideration: 1) Caprini, 2) VTE, 3) CCI, 4) ASA, 5) VES-13, and 6) Fragility. Models were then iteratively refined by eliminating non-significant predictors until the optimal model contained only significant variables. This approach was justified because including unnecessary predictors - given the high level of missing data - reduced the sample size available for model estimation, negatively affecting estimation quality.

Initial logistic regression models incorporated the clinical outcome of interest (prolonged hospitalization, 30−day serious complications, or 90−day functional decline) and six explanatory variables representing the risk assessment instruments: Caprini, VTE, CCI, ASA, VES−13, and mFI−5. Given the substantial missing data across participating centers, particularly in functional status variables and certain risk scale components, sequential model refinement through backward elimination of non−significant predictors was applied. This approach served three interconnected purposes: to reduce informational noise from excessive predictors, to mitigate sample size reduction that would result from retaining unnecessary variables with sparse data, and to enhance estimation precision. Only final reduced models containing exclusively statistically significant predictors are reported in the Results. Model selection was guided by the Akaike Information Criterion (AIC) and pseudo−R² measures (McFadden’s R² and Nagelkerke’s R²) to identify the most parsimonious and informative specification.

## Results

Comparisons between oncological and non-oncological groups revealed a significantly higher proportion of males among oncological group (62% versus 43%, p for χ² test < 0.001), as well as a greater share of current and former smokers (totaling 53% versus 29%, p < 0.001), and a higher frequency of previous malignancies (37% versus 25%, p = 0.010). Interestingly, oncological group was slightly more likely to report no family history of cancer (83%) than non-oncological group (71%), but this potential effect exceeded the adopted significance threshold (p = 0.043). Oncological patients were, on average, approximately one year older, although this result was on the border of statistical significance (p for Welch’s t-test = 0.012).

Descriptive statistics for the sample regarding patient status and risk factors are presented in **Tables 1A**-**C**, **2A**, **B**. A slight predominance of male participants was noted (51.2% of the total sample). The mean age was 84 years, with a right-skewed distribution (skewness coefficient: 0.93), indicating that most respondents were aged 80–83 years (half of the sample falls within this range), alongside a small number of individuals in advanced old age, substantially older than the mean (maximum patient age in the cohort was 97 years).

**Table 1A:** Sample characteristics: gender and variables describing patient status and risk factors - overall statistics.

Overall statistics			
Variable	Category	N	%
Gender	female	217	48.8%
	male	228	51.2%
	Total	445	100%
	Missing	2	
Independance	independent	324	73.3%
	assisted living	93	21.0%
	dependent	25	5.7%
	Total	442	100%
	Missing	5	
Sarcopenia	no	418	95.0%
	yes	22	5.0%
	Total	440	100%
	Missing	7	
Food intake	gastrostomy	9	2.1%
	feeding tube	7	1.6%
	with help	38	8.8%
	independently	380	87.6%
	Total	434	100%
	Missing	13	
Polipharmacy	0	27	6.6%
	≤3	109	26.6%
	>3	274	66.8%
	Total	410	100%
	Missing	37	
Physical activity	no	113	27.9%
	limited	140	34.6%
	yes	152	37.5%
	Total	405	100%
	Missing	42	
Alcohol	no	319	94.1%
	yes	20	5.9 %
	Total	339	100%
	Missing	108	
Smoking	no	237	59.8%
	yes, in the past	127	32.1%
	yes, currently	32	8.1%
	Total	396	100%
	Missing	51	
Oncological group	no	227	51.9%
	yes	210	48.1%
	Total	437	100%
	Missing	10	
Oncological family history	no	156	77.2%
	yes	46	22.8%
	Total	202	100%
	Missing	245	
Oncological disorders currently (other than ORL)	no	390	90.5%
	yes	41	9.5%
	Total	431	100%
	Missing	16	
Oncological disorders in past	no	274	69.7%
	yes	119	30.3%
	Total	393	100%
	Missing	54	

**Table 1B:** Sample characteristics: gender and variables describing patient status and risk factors, including stratification by oncological and non-oncological patients.

Variable	Category	Oncological group		Non-oncological group		Significance & effect size
		N	%	N	%	
Gender	female	79	38%	130	57%	χ² =16.879 Cramer’s V = 0.197 p<0.001
	male	131	62%	97	43%	
	Total	210	100%	227	100%	
Independence	independent	150	72%	169	74%	χ²= 2.806 Cramer’s V = 0.080 p=0.246
	assisted living	49	24%	42	19%	
	dependent	9	4%	16	7%	
	Total	208	100%	227	100%	
Sarcopenia	no	196	95%	214	95%	χ²= 0.040 Cramer’s V = 0.010 p= 0.841
	yes	11	5%	11	5%	
	Total	207	100%	225	100%	
Food intake	gastrostomy	6	3%	3	1%	χ²= 2.860 Cramer’s V = 0.082 p= 0.414
	feeding tube	5	2%	2	1%	
	with help	17	8%	21	10%	
	independently	181	87%	193	88%	
	Total	209	100%	219	100%	
Polipharmacy	0	17	9%	10	5%	χ²= 2.810 Cramer’s V = 0.084 p= 0.245
	≤3	49	25%	60	29%	
	>3	130	66%	137	66%	
	Total	196	100%	207	100%	
Physical activity	no	55	28%	55	27%	χ²=3.593 Cramer’s V = 0.095 p= 0.166
	limited	74	38%	64	31%	
	yes	64	33%	86	42%	
	Total	193	100%	205	100%	
Alcohol	no	150	93%	164	95%	χ²= 0.394 Cramer’s V = 0.034 p=0.530
	yes	11	7%	9	5%	
	Total	161	100%	173	100%	
Smoking	no	89	47%	142	71%	χ²= 24.786 Cramer’s V = 0.252 p= <0.001
	yes currently	20	10%	12	6%	
	yes in past	82	43%	45	23%	
	Total	191	100%	199	100%	
Oncological family history	no	87	83%	68	71%	χ²= 4.108 Cramer’s V = 0.143 p=0.043
	yes	18	17%	28	29%	
	Total	105	100%	96	100%	
Oncological disorders currently (other than ORL)	no	171	85%	213	96%	χ²= 13.489 Cramer’s V = 0.178 p=<0.001
	yes	30	15%	10	4%	
	Total	201	100%	223	100%	
Oncological disorders in past	no	121	63%	147	75%	χ²=6.583 Cramer’s V = 0.131 p=0.010
	yes	70	37%	48	25%	
	Total	191	100%	195	100%	

**Table 1C:** Sample characteristics of age including stratification by oncological vs non-oncological groups.

Age		
Characteristic	Oncological group	Non-oncological group
N	210	227
Mean ± SD (years)	84.87 ± 3.75	84.00 ± 3.40
Median (IQR), years	84 (82–87)	83 (81–86)
Variance	14.059	11.566
Minimum –Maximum, years	79 - 97	80 - 94

SD, standard deviation; IQR, interquartile range; N, number of observations.

**Table 2A:** Descriptive statistics for Caprini, CCI, ASA and mFI-5 scales – overall statistics.

Statistic	Caprini	CCI	ASA	mFI-5
N	385	317	289	245
Missing	62	130	158	202
Median (IQR)	5 (4–7)	6 (5–8)	3 (2–3)	2 (1–3)
Minimum–Maximum	3–18	1–16	1–5	0–5
Skewness	1,671	1,012	0,169	0,646
Kurtosis	5,311	1,452	0,921	-0,153

CCI- Charlson Comorbidity Index; ASA,- American Society of Anesthesiologists Physical Status Classification; IQR- interquartile range; SD- standard deviation; N- number of observations.

**Table 2B:** Descriptive statistics for VTE and VES-13 scales – overall statistics.

VTE			VES-13		
Variable	N	%	Variable	N	%
low risk (1-2p)	1	0%	<3	56	20%
moderate risk (3-4p)	112	26%	>=3	224	80%
high risk (>=5p)	311	73%	Total	280	100%
Total	424	100%	Missing	167	
Missing	23				

VTE- venous thromboembolism; VES−13- Vulnerable Elders Survey−13.

The majority of patients functioned independently; approximately one-eighth required assistance with feeding, via caregiver, nasogastric tube, or gastrostomy. Most respondents (95%) did not present with sarcopenia. Two-thirds of participants reported taking more than three medications. Only one in twenty admitted to alcohol consumption, though just 60% had never smoked tobacco. Currently, 8% of respondents were active smokers. Over one-third of participants were physically active.

Nearly half of the sample consisted of oncological patients. At the time of assessment, nearly one in ten individuals (41 of 431 observations, 9.5%) suffered from malignancies other than otolaryngological cancers, while 30% had a history of cancer (119 of 393, 30.3%), and nearly one-fourth had family members who had experienced malignancies (46 of 202, 22.8%).

The frequency of prolonged hospitalizations did not differ significantly between oncological and non-oncological groups (χ²(1) = 3.850, p = 0.050, exceeding the adopted significance threshold), although prolonged hospitalizations were numerically more common among oncological group (61.2% versus 38.8% among non-oncological group).

Among 375 cases, 30-day serious complications were detected in 17 instances (4.5%), and among 287 cases, 90-day functional decline was reported in 28 instances (9.8%). No relationship was found between these outcomes and oncological status (in both cases, p for χ² test > 0.10). However, the risk of 30-day serious complications was associated with the presence of non-otolaryngological cancer (3 of 35 cases, 14.3%, in the affected group versus 12 of 332 cases, 3.6%, in the unaffected group, p = 0.004). Sample characteristics—age, sex, and variables describing patient status and risk factors, including stratification by oncological and non-oncological groups are shown in **Tables 1A–C**, **2A**, **B**.

### Outcome characteristics according to applied risk scales

**Tables 3A**, **B** provides detailed descriptive statistics for the scales used to assess patient risk and health status (Caprini score, Charlson Comorbidity Index (CCI), American Society of Anesthesiologists scale (ASA), Venous Thromboembolism Risk Assessment Tool (VTE), Vulnerable Elders Survey-13scale (VES-13) and the modified 5-item frailty index (mFI-5) hereinafter referred to as Fragility scale). Notably, a large proportion of the sample was assigned high values on the Caprini score and VTE risk scales, both of which are used to evaluate the risk of thrombosis. As many as 73% of patients were classified as high risk according to the VTE risk scale, and both the mean and median Caprini score was 5 (a value of 5 or greater indicates a very high risk). Patients also recorded high values on the CCI (mean CCI was 6), as well as on the ASA (mean score was 3, indicating significant chronic disease limiting patient performance or activity) and VES-13 scales (80% of patients achieved a score of ≥3, reflecting high risk for loss of independence).

**Table 3A:** Descriptive statistics for Caprini, CCI, ASA, mFI-5 scales including stratification by oncological and non-oncological patients.

Scale	Statistic	Oncological patients	Non-oncological patients	p
Caprini	N	184	195	<.001
	Median (IQR)	6 (5–7)	5 (4–6)	
	Mean ± SD	6.4 ± 2.03	5.36 ± 2.07	
CCI	N	145	168	<.001
	Median (IQR)	7 (6–8)	5 (4–6)	
	Mean ± SD	7.35 ± 2.17	5.67 ± 1.77	
ASA	N	152	132	<.001
	Median (IQR)	3 (2–3)	3 (2–3)	
	Mean ± SD	2.74 ± 0.56	2.51 ± 0.55	
mFI-5	N	112	128	0.774
	Median (IQR)	2 (1–3)	2 (1–3)	
	Mean ± SD	1.99 ± 1.12	2.01 ± 1.03	

CCI- Charlson Comorbidity Index; ASA,- American Society of Anesthesiologists Physical Status Classification; IQR- interquartile range; SD- standard deviation; N- number of observations.

**Table 3B:** Descriptive statistics for VTE and VES-13 scales including stratification by oncological and non-oncological patients.

Scale	Variable	Oncological patients		Non-oncological patients			p
		N	%		N	%	
VTE	low risk (1-2p)	0	0%		1	0%	<.001
	moderate risk (3-4p)	26	13%		85	40%	
	high risk (>=5p)	176	87%		129	60%	
	Total	202	100%		215	100%	
VES-13	<3	25	19%		30	21%	0.673
	>=3	107	81%		113	79%
	Total	132	100%		143	100%

VTE- venous thromboembolism; VES−13- Vulnerable Elders Survey−13.

Compared to non-oncological group, oncological group had significantly higher mean scores on the Caprini scale (mean of 6.4 versus 5.4 for non-oncological group; p < 0.001 in both t and U tests—providing strong evidence for differences in means and stochastic dominance of the oncological group), CCI (mean of 7.4 for oncological group versus 5.7 for non-oncological group; p < 0.001), ASA (mean of 2.743 for oncological and 2.515 for non-oncological group; p ≤ 0.001), and VTE (87% classified as high risk among oncological group compared to 60% among non-oncological group; p for χ² test < 0.001). Importantly, the difference in CCI risk was of large effect size (Cohen’s |d| = 0.847), while for the Caprini score it was moderate (Cohen’s |d| = 0.505), moderate for VTE (Cramer’s V = 0.307), and low to moderate for ASA (Cohen’s |d| = 0.414).

No statistically significant differences were observed for the VES-13 and Fragility scale (p > 0.62).

**Table 4** provides logistic regression models for the risk of prolonged hospitalizations, 30-day serious complications, and 90-day functional decline in oncological and non-oncological groups.

**Table 4:** Coefficients and quality measures of logistic regression models explaining the risk of prolonged hospitalizations, 30-day serious complications, and 90-day functional decline.

Group	Non-oncological group		Oncological group	
Variable	Intercept	Caprini	Intercept	CCI
Estimate	-4.312	0.406	-3.487	0.321
Robust SE	0.641	0.099	0.734	0.088
Standardized^+^	-2.137	0.842	-1.129	0.696
*Z*	-6.723	4.084	-4.752	3.646
*P*	<.001	<.001	<.001	<.001
OR 95% CI	(0.004; 0.047)	(1.235; 1.824)	(0.007; 0.129)	(1.160; 1.637)
Nagelkerke *R²*	0.185		0.125	
Δχ² (p)	20.279 (<.001)		12.976 (<.001)	
AIC	133.075		157.835	

Robust SE-robust standard error; OR 95% CI-95% confidence interval of odds ratio; R²N- Nagelkerke’s pseudo-R2; df- degrees of freedom; AIC-Akaike information criterion; null model-intercept-only model, used as a benchmark.

^+^ Standardized estimates represent estimates where the continuous predictors are standardized (X- standardization).

The results indicate that, for non-oncological group, the Caprini scale most accurately explained the risk of PH, 30-day serious complications, and 90-day functional decline (p < 0.001). The corresponding model achieved very high quality indices (McFadden’s R² = 0.136, Nagelkerke’s R² = 0.185). In contrast, for oncological group, the best risk predictor was the CCI scale (p < 0.001) (McFadden’s R² = 0.078, Nagelkerke’s R² = 0.125). Interestingly, using the Caprini scale as the sole explanatory variable for oncological group resulted in a non-significant model (p = 0.132), and a similar effect was observed when using the CCI as the only predictor for non-oncological group (p = 0.107). Exact coefficients and model quality indices are reported in **Table 4**. The summary of group differences and key predictors are presented in the **Table 5**.

**Table 5:** Summary of group differences and key predictors.

Parameter	Oncological group	Non-oncological group	p-value	Effect size
Male gender	62%	43%	<0.001	Cramer’s V = 0.197
Current/past smoking	53%	29%	<0.001	Cramer’s V = 0.252
Prior malignancy	37%	25%	0.010	Cramer’s V = 0.131
High VTE risk	87%	60%	<0.001	Cramer’s V = 0.307
Mean CCI	7.4	5.7	<0.001	Cohen’s
Best predictor (logistic regression)	CCI	Caprini	<0.001	–

## Discussion

The presented results indicate that oncological group, as compared to non-oncological group, exhibit a markedly different demographic and clinical profile, as well as perioperative risk factors and early postoperative outcomes.

Our key findings showed that oncological patients were much more likely to be male, current or former smokers, and to have a personal history of malignancy. These differences were statistically robust and accompanied by moderate to large effect sizes, underlining substantial heterogeneity between groups, especially in relation to modifiable risk factors associated with cancer incidence. Alcohol consumption was rare overall, but both current and former smoking were significantly more prevalent among oncological group.

Compared with published literature, our results align closely with established epidemiological and clinical patterns observed in elderly patients with head and neck cancers. Notably, several international studies and meta-analyses confirm the marked predominance of males and smokers among oncological populations, with men experiencing a two- to eleven-fold higher incidence for HNC. The prevalence of smoking as a risk factor, both current and former, is reliably elevated in oncological groups compared with general or control populations, as is documented in large cohort studies and pooled analyses from Asia and Europe (20).

Numerous studies have demonstrated that hazardous alcohol use, impaired baseline functional status, and sarcopenia are powerful predictors of postoperative complications, functional decline and mortality in older surgical and head and neck cancer populations. Our intention in the discussion is not to challenge this well established evidence, but to describe the specific pattern observed in our cohort, in which alcohol consumption was infrequent, baseline functional status was relatively preserved, and clinically overt sarcopenia was rare, and therefore these factors did not emerge as major discriminators between oncological and non oncological groups nor as dominant predictors in our multivariable models (21–23).

Age distributions revealed a subtle trend towards higher mean age in the oncological group; however, this finding hovered at the threshold of statistical significance, suggesting only a marginal difference. With respect to age, international epidemiological data also demonstrate a right-skewed distribution with higher mean and median ages for cancer diagnosis in high-income countries, which matches the subtle age trend documented in our cohort (24, 25).

Numerous large surgical and oncological series have shown that higher CCI scores are strongly associated with increased postoperative morbidity, prolonged length of stay and long-term mortality in older and cancer populations, including head and neck cancer patients. Likewise, the Caprini risk score has been repeatedly validated as a robust predictor of venous thromboembolism and related complications, allowing identification of a more than 10 fold gradient of VTE risk across surgical risk categories (26, 27).

The VTE Risk Assessment Tool, VES-13 and mFI-5 have been shown in previous work to be associated with adverse outcomes in older and oncogeriatric populations, including higher risks of postoperative complications, functional decline, prolonged hospital stay and mortality (28, 29).

In our study the vast majority of our cohort was already classified as high risk on the VTE Risk Assessment Tool, which likely produced a ceiling effect and limited its discriminative power beyond what was already captured by the Caprini score. The VES-13 results, noting that although approximately 80% of patients met the conventional vulnerability threshold (≥3 points), this high prevalence compressed the score distribution and may explain why VES-13 did not outperform CCI or Caprini in predicting our composite “Problems” endpoint, despite its well documented association with functional deterioration and mortality in other surgical series. The mFI-5 frailty scores were relatively low and showed limited variability in this selected 80+ group, which is consistent with reports that mFI 5 is most powerful when there is a broad frailty spectrum, and may lose predictive accuracy when applied to highly preselected, comparatively robust elderly patients.

The Caprini score was originally designed as a venous thromboembolism specific risk assessment model rather than a global surgical risk index. In our paper, however, we did not a priori select Caprini as “the best” predictor of adverse outcomes; instead, it emerged as the single most informative scale in the multivariable models for non oncological group when all six instruments (Caprini, CCI, ASA, VTE, VES 13, frailty) were entered simultaneously and non significant variables were stepwise removed (30, 31).This finding is biologically and clinically plausible. Venous thromboembolism and related medical complications are well recognized contributors to postoperative morbidity, prolonged length of stay and mortality in surgical patients, and multiple large validation studies have shown that the Caprini score discriminates a 10 to 15 fold gradient of VTE risk across surgical populations, including otolaryngology–head and neck surgery. Given that our composite “Problems” endpoint explicitly included prolonged hospitalization and serious 30 day complications, it is not surprising that an index capturing cumulative VTE and medical risk factors performed well as a predictor in the non oncological subgroup (31).

In our cohort, in non oncological 80+ patients, Caprini emerged as the best single predictor of the composite “Problems” endpoint, which is consistent with its original purpose as a VTE centered risk model and with data showing that thromboembolic and medical complications are key drivers of adverse early outcomes in frail surgical patients. In contrast, among oncological group the CCI was the strongest predictor, suggesting that in this very old HNC population the global comorbidity burden, notably cardiovascular and metabolic disease captured by CCI, may outweigh procedure specific thrombotic risk as a determinant of complications, prolonged hospitalization and functional decline (32).

We highlight that the divergence between Caprini (non oncological group) and CCI (oncological group) supports the notion that risk stratification tools should be tailored to the underlying disease context. In oncological patients, the Charlson Comorbidity Index rather than Caprini was the strongest predictor, highlighting the predominant role of global multimorbidity in this cohort, whereas in non oncological admissions the thromboembolic/medical risk profile captured by Caprini appeared more salient (8, 32, 33).

In other words, while both indices capture overlapping dimensions of vulnerability, CCI appears to integrate better the chronic multi morbid profile typical of octogenarian HNC patients, whereas Caprini more accurately reflects the acute perioperative thromboembolic risk profile in non oncological ENT admissions (34).

The absence of strong independent associations for the VTE tool, VES 13 and mFI 5 in our multivariable models should be interpreted as a function of sample characteristics (high baseline risk and restricted score variability) and limited power, rather than as evidence against their usefulness. Our data support a pragmatic approach in which CCI and Caprini are prioritized for risk stratification in this specific setting, while VES-13 and mFI-5 remain essential components of comprehensive geriatric assessment that are well supported by the broader literature (18, 19, 35).

Our analysis of risk scales demonstrated that oncological group consistently scored higher on Caprini, CCI, ASA, and VTE, metrics strongly linked to complications and adverse outcomes in elderly surgical populations. Critically, nearly 90% of our oncological patients were classified as high risk for thromboembolic events, and both comorbidity burden and anesthetic risk were significantly elevated.

Despite the more adverse risk profile, the frequency of prolonged hospitalizations did not differ significantly between groups, though oncological patients did experience numerically more such events. Similarly, neither 30-day serious complications nor 90-day functional decline showed significant associations with oncological status, except for a markedly higher complication rate among patients with non-head-and-neck cancer diagnoses. Finally, our report of similar rates of prolonged hospitalization and functional decline between groups, despite clear risk stratification differences, finds support in contemporary analyses which suggest that well-coordinated perioperative care and selection mitigate these risks even in high-risk geriatric oncology patients (36).

The main strengths of this study are its multicenter design, the relatively large and well-characterized cohort of patients aged ≥80 years, and the multidimensional assessment combining comorbidity, thromboembolic, anesthetic, frailty and functional measures, including a direct comparison of oncological and non−oncological patients in the octogenarian group, which is poorly represented in the existing literature. Despite the strengths of multicenter design employed, our study has several limitations. The main limitations of this study relate to missing data and variability in data collection across centers. In addition, the exploratory nature of the analysis with multiple comparisons increases the risk of Type I error, although this was mitigated by using a stricter significance level. Another limitation is the absence of detailed adjustment for the timing and toxicity of adjuvant radiotherapy in relation to the 90-day functional decline endpoint and it should be specifically addressed in future studies.

In summary, our results are strongly corroborated by recent and high-impact literature, highlighting the interplay of gender, modifiable lifestyle factors, comorbidity burden, and tailored risk assessment in elderly patients with HNC. These findings emphasize the ongoing need for individualized preoperative evaluation and risk stratification supported by validated predictive tools (37). Our research emphasizes the importance of a comprehensive geriatric assessment that considers functional status, comorbidities, and frailty, rather than age alone, for optimal patient selection and perioperative care. Individualized surgical management in suitable elderly patients offers an effective therapeutic option for HNC and can be well tolerated when risks are appropriately addressed.

In summary, well-planned oncological surgery in octogenarians may represent an effective and justified treatment modality, provided there is specialized preoperative assessment, perioperative multidisciplinary management, and active risk mitigation tailored to the older adult population.

## Data Availability

The raw data supporting the conclusions of this article will be made available by the authors, without undue reservation.
